# Late diagnosis of subcoracoid type 6 AC dislocation: A case report

**DOI:** 10.1051/sicotj/2019036

**Published:** 2019-10-25

**Authors:** Mehmet Kapicioglu, Huzeyfe Cetin, Kerem Bilsel

**Affiliations:** 1 Bezmialem Vakif University, School of Medicine, Department of Orthopaedics and Traumatology Vatan Cd Fatih 34093 İstanbul Turkey

**Keywords:** Acromioclavicular dislocation, Subcoracoid, Delayed, Type 6

## Abstract

Acromioclavicular (AC) dislocation is a common type of shoulder injury. Although the incidence of acromioclavicular dislocation is frequent, there are different opinions regarding the treatment. Many different techniques have been proposed for the surgical treatment of AC dislocations, but all these methods have been questioned from different angles, and the gold standard in terms of treatment has not yet been determined. There are six types described by Rockwood et al. and type 6 has two types: subacromial and subcoracoid. Subcoracoid AC Type 6 dislocations are seen very rarely and difficult to diagnose in initial clinical findings or can be simply overlooked due to associated more serious injuries which take more attention. The mechanism of injury of a type 6 AC dislocation is hyperabduction and external rotation of the shoulder. A small number of type 6 subcoracoid AC dislocations have formerly been reported and apart from one case all of them were acutely diagnosed and treated with open reduction and internal fixation. In this paper, we report a case of late diagnosis of subcoracoid type 6 AC dislocation, along with its rare and previously unreported surgical management.

## Introduction

Acromioclavicular (AC) dislocation is a common type of shoulder injury. Although the incidence of acromioclavicular dislocation is frequent, there are different opinions regarding the treatment [1, 2]. Many different techniques have been proposed for the surgical treatment of AC dislocations, but all these methods have been questioned from different angles, and the gold standard in terms of treatment has not yet been determined [2]. There are six types described by Rockwood et al. and type 6 has two types: subacromial and subcoracoid [2]. Subcoracoid AC type 6 dislocations are seen very rarely and difficult to diagnose in initial clinical findings or can be simply overlooked due to associated more serious injuries which take more attention [2]. The mechanism of injury of a type 6 AC dislocation is hyperabduction and external rotation of the shoulder [2, 3]. A small number of type 6 subcoracoid AC dislocations have formerly been reported, and apart from one case, all of them were acutely diagnosed and treated with open reduction and internal fixation [1–11]. In this paper, we report a case of late diagnosis of subcoracoid type 6 AC dislocation, along with its rare and previously unreported surgical management.

## Case report

A 40-year old male laborer applied to our clinic with a complaint of sustained pain on his right shoulder and an inability to move his right shoulder after being injured in an in-vehicle car accident. According to his anamnesis, he was diagnosed with second and third rib fractures and pneumothorax that were initially treated at a thoracic surgery department for 6 weeks. After he was discharged, he applied to our clinic with right shoulder pain. We discovered that an AC dislocation type 6 had been overlooked when he first admitted to emergency department. There wasn’t a relevant family or medical history.

On physical examination, we observed that the patient’s right shoulder was swollen and deformed. There was local tenderness on the AC joint but no neurological deficits in the right shoulder or arm. Active range of motion (ROM) of the right shoulder also could not be determined due to pain. The patient was keeping his right shoulder internally rotated; there was a restriction during passive internal rotation, and passive external rotation was very painful.

**Figure 1:**
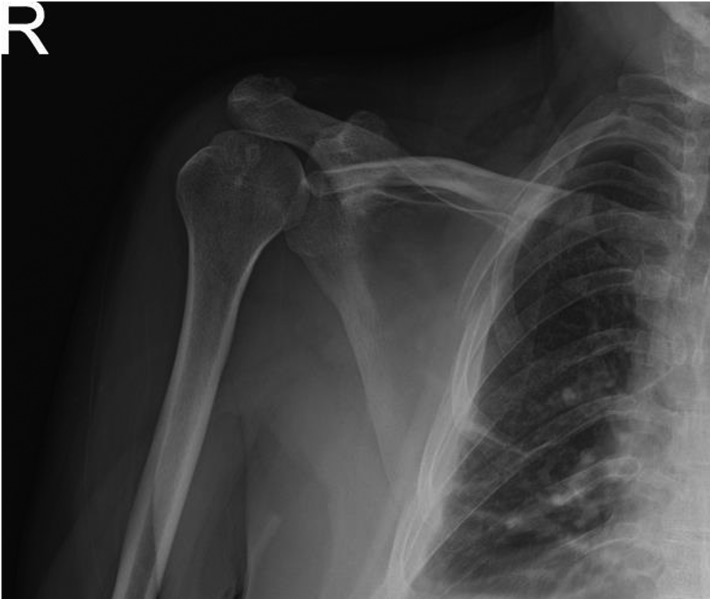
AP radiographs of the right shoulder 8 weeks after injury demonstrating subcoracoid dislocation of the distal clavicle.

**Figure 2:**
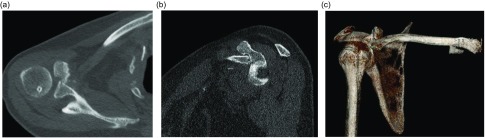
(a) Axial, (b) sagital, and (c) 3D computed tomography images of the right shoulder 8 weeks after injury demonstrating subcoracoid dislocation of the distal clavicle.

Radiographs showed an inferiorly displaced distal clavicle (Figure 1). Computed tomography demonstrated subcoracoid AC dislocation type 6 (Figure 2). The distal clavicle was in front of the glenoid and blocking the humeral head. Nondisplaced and healed right scapular body fractures and multiple rib fractures were also spotted.

**Figure 3:**
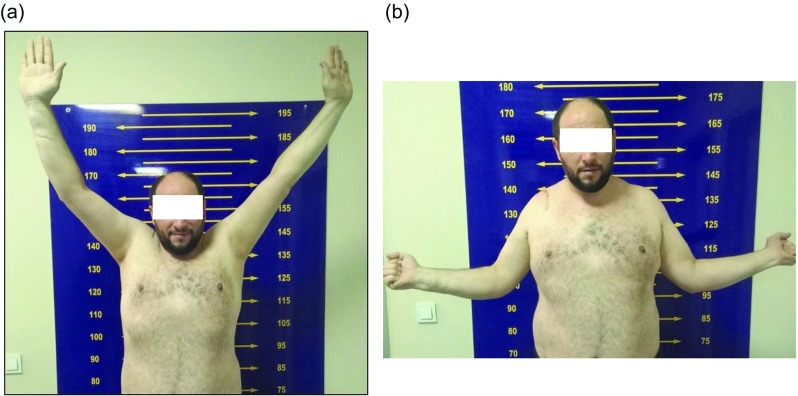
(a and b) Postoperative physical examination show good results.

**Figure 4:**
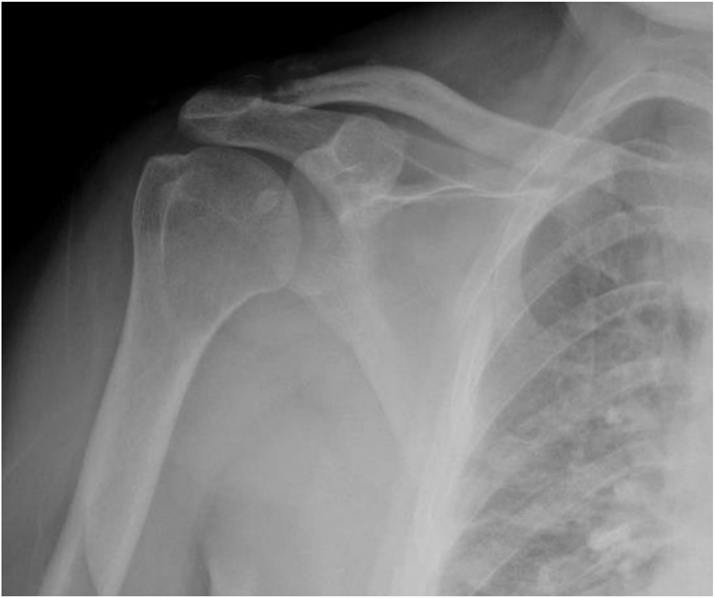
Final radiographs at 1 year showed good reduction of AC joint with minimal ossification, but no calcification or bony bar imaging or joint space narrowing.

Six weeks after the accident, we performed open reduction and internal ligament and capsular repair of the AC joint. The patient was placed in a beach chair position. An inverted “J” incision was made extending from posteromedial corner of AC joint to the lateral border of coracoid process. The deltoid and trapezius muscles were found to have been stripped off the lateral end of the clavicle. In addition, the acromioclavicular ligaments were disrupted. The fibrotic soft tissue in acromioclavicular joint was removed. Conjoint tendon was intact; the distal clavicle was felt under the coracoid process. Coracoacromial and coracohumeral ligaments around coracoid process were cut, and pectoralis minor tendon was released from the medial border of the coracoid. The distal clavicle was removed under coracoid process by releasing the soft tissue. By abducting the shoulder, the lateral traction of the scapula was done. Thus, the distal clavicle was reduced into its anatomical position. After the reduction, because of the osseous disorganization related to the clavicular osteolysis, the distal tip of clavicle was excised about 5 mm. Because of the inferior displacement and a 6-week injury, coracoclavicular ligaments could not be evaluated clearly. There were no coracoclavicular ligament parts that could be repaired. With the help of the K-wire, four bone tunnels were opened in both acromion and distal clavicles. Internal bracing was performed for acromioclavicular joint with No: 2 ethibond^©^ sutures (Ethicon LLC/US) [12]. Finally, the deltotrapezial fascia was attentively repaired over the top of the clavicle. After the incision was closed, the upper extremity was placed on an arm sling. Postoperatively, shoulder movement was restricted but wrist and elbow movement was possible. Passive ROM exercises were initiated 4 weeks after surgery. Active ROM exercises were initiated at fifth week after surgery, and stretching and strengthening exercises were prescribed as a home program at the second month, postoperatively. At final follow-up first year postoperatively, he had no pain when moving his injured shoulder, and his motion was similar to the other shoulder (forward elevation, 175°/175°; internal rotation to T10, and external rotation, 60°/60°; bilaterally; Figure 3). Final radiographs 1 year after the surgery showed good reduction of AC joint with minimal ossification, but no calcification or bony bar imaging (Figure 4). At that time, the patient returned to his previous daily activities and his job with pain-free right shoulder movement.

## Discussion

Subcoracoid AC type 6 dislocation is excessively rare trauma, and the injury is frequently associated with different injuries [9]. The mechanism of subcoracoid dislocation of the clavicle includes forceful abduction and external rotation of the shoulder [5]. Subcoracoid AC joint dislocations may be overlooked because they are seldom encountered and often accompanied by other serious injuries. For this reason, it is important to be aware of the possibility of AC joint injury in all multi-trauma patients with shoulder symptoms. Undertaking dedicated shoulder and AC joint imaging in all such patients is recommended [2]. In our case, we reported a subcoracoid AC joint dislocation was diagnosed with a delay of 2 months. However, despite this delay in diagnosis and treatment, it is seen that the result is promising.

It is quite remarkable that considering the typically high-energy mechanism of this injury and the risk of brachial plexus and axillary vessels compression because of the narrowed subcoracoid space, there is no report of vascular or persistent neurological damage in subcoracoid type 6 AC dislocation cases [7]. Nevertheless, temporary neurologic dysfunction has been reported [2, 5, 9].

Previously, very few isolated subcoracoid type 6 AC dislocations have been reported. Most of them have been treated by open reduction and internal fixation with different techniques including coracoclavicular screw, acromioclavicular K-wire fixation, tension band wiring, or coracoclavicular suture repair [1, 4–8, 10, 11]. Surgical techniques usually focus on coracoclavicular ligament augmentation because it has been shown to be the primary stabilizer of the acromioclavicular joint [4]. Gerber and Rockwood reported the use of temporary coracoclavicular lag screw and acromioclavicular K-wire, ligament repair, and imbrication of the deltotrapezial fascia over the top of the clavicle [5]. Patterson reported the use of acromioclavicular Steinmann pins to aim stable fixation of AC joint [8]. Torrens et al. stabilized the clavicle with a coracoclavicular screw as described by Bosworth [10]. Canbora et al. reported that they undertook coracoclavicular reconstruction with coracoacromial ligament transfer [4]. Emami et al. reported that they provided stabilization with coracoclavicular screw and tension band wiring [1]. Wisniewski reported acromioclavicular K-wire fixation, and wires have been removed 6 weeks after surgery [11]. Differently, in only two cases previously reported in the literature, only open reduction was performed and no implant or suture material was used. McPhee reported an open reduction without any fixation method. Stabilization is achieved by suturing deltotrapezial fascia and joint capsule [6]. In an isolated subcoracoid type 6 AC dislocation reported by Neumann et al., soft tissue repair was performed without any fixation method following open reduction, and a functionally satisfactory result was reported at 8 years of follow-up [7].

In our particular case, open reduction was performed, and the distal clavicle was resected to prevent the development of secondary degenerative changes due to the injury to the AC joint and its intra-articular disc. Acromioclavicular ligament and capsule were repaired by using non-absorbable Ethibond^©^ No: 2 suture (Ethicon US, LLC). Dorsal soft tissue was sutured onto capsule and acromioclavicular ligaments. No special coracoclavicular ligament repair was performed. No specific implant or anchor was used to fix AC joint. Thus, a dynamic AC joint fixation was provided instead of rigid fixation. There was no need for reoperation for implant removal since no temporary fixation material was used. Therefore, the risks of reoperation were avoided, and the costs were reduced while eliminating the risk of neurovascular injury during implant placement.

Previous reports have documented different radiographic changes including coracoclavicular ossification [5, 6], bony bar around the AC joint [1, 5], narrowing of the joint space [3, 5, 11] and osteolysis of the distal end of clavicle [5, 10] during long-term follow-up. However, despite all these radiological findings, all patients had satisfactory functional results. In our case, final radiographs 1 year after the surgery showed good reduction of AC joint with minimal ossification, without any calcification, bony bar imaging or joint space narrowing. This result may be achieved thanks to the fact that no coracoclavicular or acromioclavicular implant was used, and distal clavicle resection was performed during the surgery.

## Conclusion

Subcoracoid AC joint dislocation is a very rare shoulder girdle injury. The diagnosis may be delayed and shadowed due to major injuries. Open reduction and internal fixation are recommended for the treatment. Non-rigid, dynamic acromioclavicular internal bracing with simple suture may be sufficient for fixation. With good surgical technique and adequate rehabilitation, promising results can be obtained even in cases of late diagnosis and treatment.
